# Expansion of fibroblast cell sheets using a modified MEEK micrografting technique for wound healing applications

**DOI:** 10.1038/s41598-022-21913-x

**Published:** 2022-11-03

**Authors:** Kanokaon Benchaprathanphorn, Phongphot Sakulaue, Wanwipa Siriwatwechakul, Pornprom Muangman, Kusuma Chinaroonchai, Nantaporn Namviriyachote, Kwanchanok Viravaidya-Pasuwat

**Affiliations:** 1 Biological Engineering Program, Faculty of Engineering, King Mongkut’s University of Technology Thonburi, Bangkok, 10140 Thailand; 2 School of Bio-Chemical Engineering and Technology, Sirindhorn International Institute of Technology, Thammasat University, Khlong Luang, 12120 Pathumthani Thailand; 3 Trauma Division, Department of Surgery, Faculty of Medicine, Siriraj Hospital, Mahidol University, Bangkok, 10700 Thailand; 4 Department of Chemical Engineering, Faculty of Engineering, King Mongkut’s University of Technology Thonburi, Bangkok, 10140 Thailand; 5 Biological Engineering and Chemical Engineering Department, Faculty of Engineering, King Mongkut’s University of Technology Thonburi, 126 Pracha Uthit Rd., Bang Mod, Thung Khru, Bangkok, 10140 Thailand

**Keywords:** Biological techniques, Biotechnology, Health care, Medical research

## Abstract

Cell sheet engineering, a scaffold-free approach to fabricate functional tissue constructs from several cell monolayers, has shown promise in tissue regeneration and wound healing. Unfortunately, these cell sheets are often too small to provide sufficient wound area coverage. In this study, we describe a process to enlarge cell sheets using MEEK micrografting, a technique extensively used to expand skin autografts for large burn treatments. Human dermal fibroblast cell sheets were placed on MEEK’s prefolded gauze without any use of adhesive, cut along the premarked lines and stretched out at various expansion ratios (1:3, 1:6 and 1:9), resulting in regular distribution of many square islands of fibroblasts at a much larger surface area. The cellular processes essential for wound healing, including reattachment, proliferation, and migration, of the fibroblasts on expanded MEEK gauze were superior to those on nylon dressing which served as a control. The optimal expansion ratio with the highest migration rate was 1:6, possibly due to the activation of chemical signals caused by mechanical stretching and an effective intercellular communication distance. Therefore, the combination of cell sheet engineering with the MEEK micrografting technique could provide high quality cells with a large coverage area, which would be particularly beneficial in wound care applications.

## Introduction

Human skin is the integument organ that performs vital roles, including as a barrier against pathogens, thermoregulation and nerve sensation. Thus, lacking skin or suffering a skin injury gives rise to many complications, including infection, tissue necrosis, hematomas, etc. Although some types of wounds or injuries repair themselves quickly, on occasion the healing can be protracted. Prolonging the healing time can increase the risks or damage when skin is lost. In recent years, scaffold-free cell sheet engineering has been shown to be a promising treatment for chronic wounds or harmful injuries by providing a high re-epithelialization rate and reducing wound closure time^1,2^. Cell sheet technology utilizes temperature-responsive polymers, such as poly (*N*-isoproprylacrylamide, PNIAM) and its derivatives, to control cell attachment and detachment by changing the temperature^3,4^. Several cell monolayers along with their deposited extracellular matrix, harvested from PNIAM-coated culture surfaces, can be stratified to form 3-dimensional tissue constructs, allowing for the fabrication of thicker tissues with more complex structures^5^. In addition, the immunological response, which is a common problem in tissue engineering, can be significantly reduced without the use of biodegradable scaffolds^6^. However, the size of the harvested cell sheet was still not adequate to cover a typical wound area, being approximately ten times larger than the current harvested cell sheets^7^. When the cell sheets are detached from the temperature-responsive culture surfaces, they undergo cytoskeletal reorganization^8^, causing cell sheet shrinkage and resulting in practically a 40% size reduction^5^. The shrinkage and reduction of the cell sheets are common problems in cell sheet engineering^9^. Using larger tissue culture dishes could overcome this problem. However, a significantly large number of cells are required for cell sheet construction, leading to a long period of cell expansion and increasing the risks and complications during hospitalization. Therefore, increasing the temperature-responsive culture surface area may not be the fabrication solution to fabricate sufficiently large cell sheets required for clinical treatment.

In the treatment of large burn wounds, skin graft meshing is often used to increase the area of the skin graft and allows exudate to escape. However, the mesh with expansion ratios above 1:6 was shown to be impractical, delaying the re-epithelialization process^10^. Due to the limited expansion ratio of skin graft meshing, a new technique called MEEK Micrografting, was introduced^11,12^. This technique can enlarge the original autologous skin graft (42 × 42 mm^2^) by several expansion ratios: 1:3, 1:4, 1:6 and 1:9. To enlarge the skin graft, a split-thickness skin graft is collected, glued onto the prefolded MEEK gauze, cut to create small square islands of skin and stretched out until the pleats become completely unfolded^13^. The expanded skin grafts at ratios of 1:3, 1:4, 1:6 and 1:9 resulted in areas of 73 × 73 mm^2^, 84 × 84 mm^2^, 102 × 102 mm^2^, and 126 × 126 mm^2^, respectively, and the spaces between each graft island were 2.20 mm, 3 mm, 4.35 mm and 6 mm, respectively^14,15^. The benefits of the MEEK Micrograft method include a higher skin graft expansion ratio, a more accurate portion of expansion than traditional mesh grafting, and ease of handling^11,12^. MEEK’s expansion ratios of 1:4–1:9 are predominantly used to treat severe burn injury, providing a high re-epithelialization rate in patients either with or without diabetic conditions in addition to reducing mortality and hospitalization^10,16–18^. Additionally, patients who received MEEK treatment have statistically low evidence of graft rejection or contamination because the skin graft lacks the skin tissue connection between skin lands^12,13^. To date, the MEEK micrografting technique has only been carried out using patients’ skin grafts and has not been used with any tissue-engineered skin substitutes or cultured skin constructs. Since tissue-engineered skin is distinctly different from real skin samples, the possibility of using the MEEK micrografting technique with skin constructs needs further investigation.

Based on these benefits, we proposed applying the modified MEEK micrografting technique to expand the harvested skin cell sheets constructed from temperature-responsive polymer surfaces, which, to the best of our knowledge, has never been investigated before. In this study, the harvested human fibroblast cell sheets were expanded at expansion ratios of 1:3, 1:6 and 1:9 by using the modified MEEK technique. The characteristics of the cell sheets after being expanded, including cell viability, reattachment capability, growth potential and migration capacity were compared with the cell sheets on typical nylon wound dressing, which was made of polyamide, similar to the gauzes used in the MEEK micrografting technique, and served as a control.

## Results

### Cell viability and reattachment of the fibroblast sheet on nylon dressing

**Figure 1:**
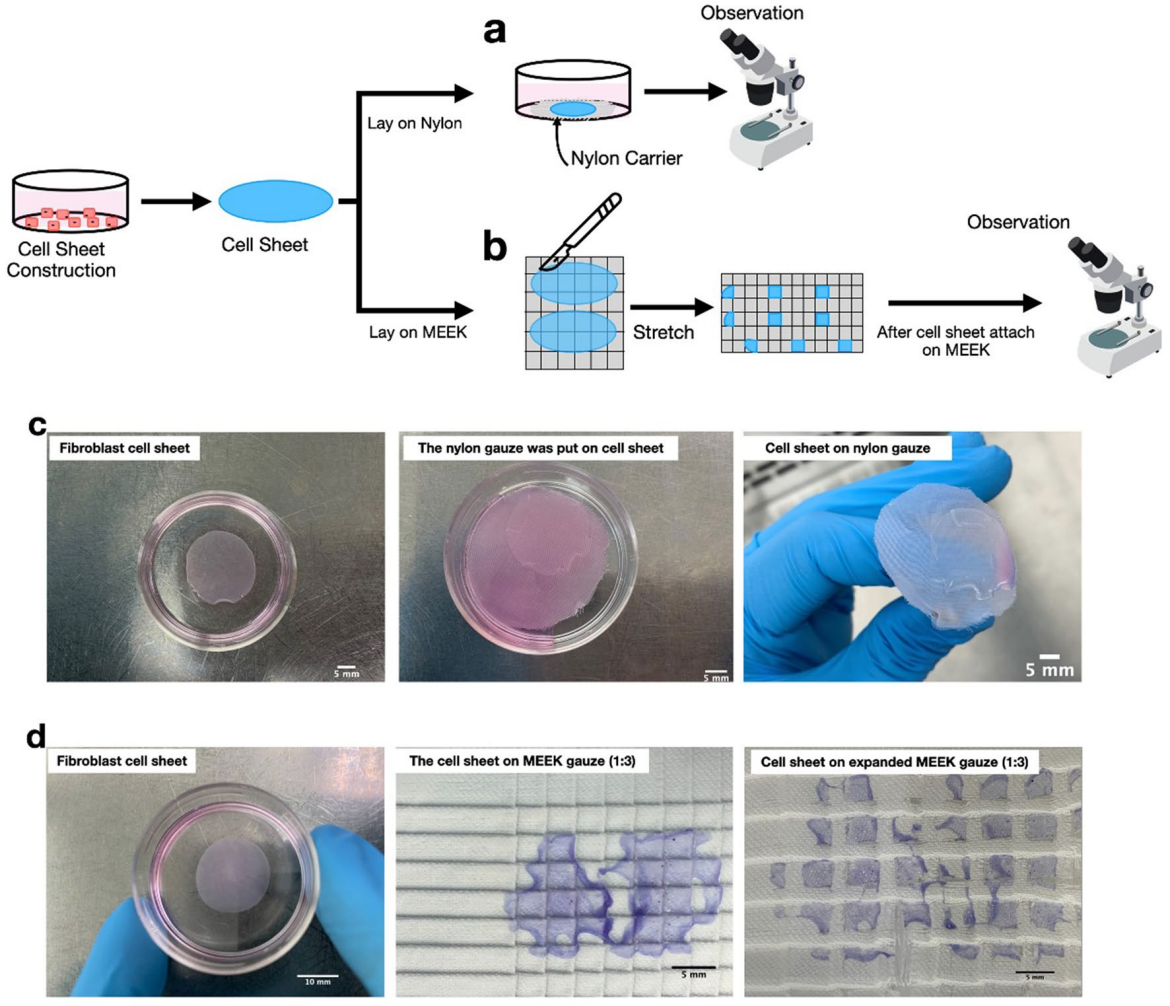
A brief schematic representation of the experimental procedure for transferring fibroblast cell sheets onto nylon dressing and MEEK gauzes. After the cell sheet was harvested from the temperature-responsive culture surface, (**a**) the sheet was immediately transferred to sterile nylon dressing before observation. Otherwise, (**b**) the cell sheet was transferred to prefolded MEEK gauze. Afterwards, the sheet was cut vertically and horizontally using a surgical blade. Then, the MEEK gauze was stretched to all sides to separate the cell sheet into many small cell islands. (**c**) The nylon dressing was overlaid on top of the fibroblast sheet before it was flipped using forceps, allowing the cell sheet to face up. (**d**) The fibroblast sheet was transferred onto the MEEK gauze before being cut and stretched, showing several square cell islands. Note that the cells were stained with trypan blue for ease of visualization.

**Figure 2:**
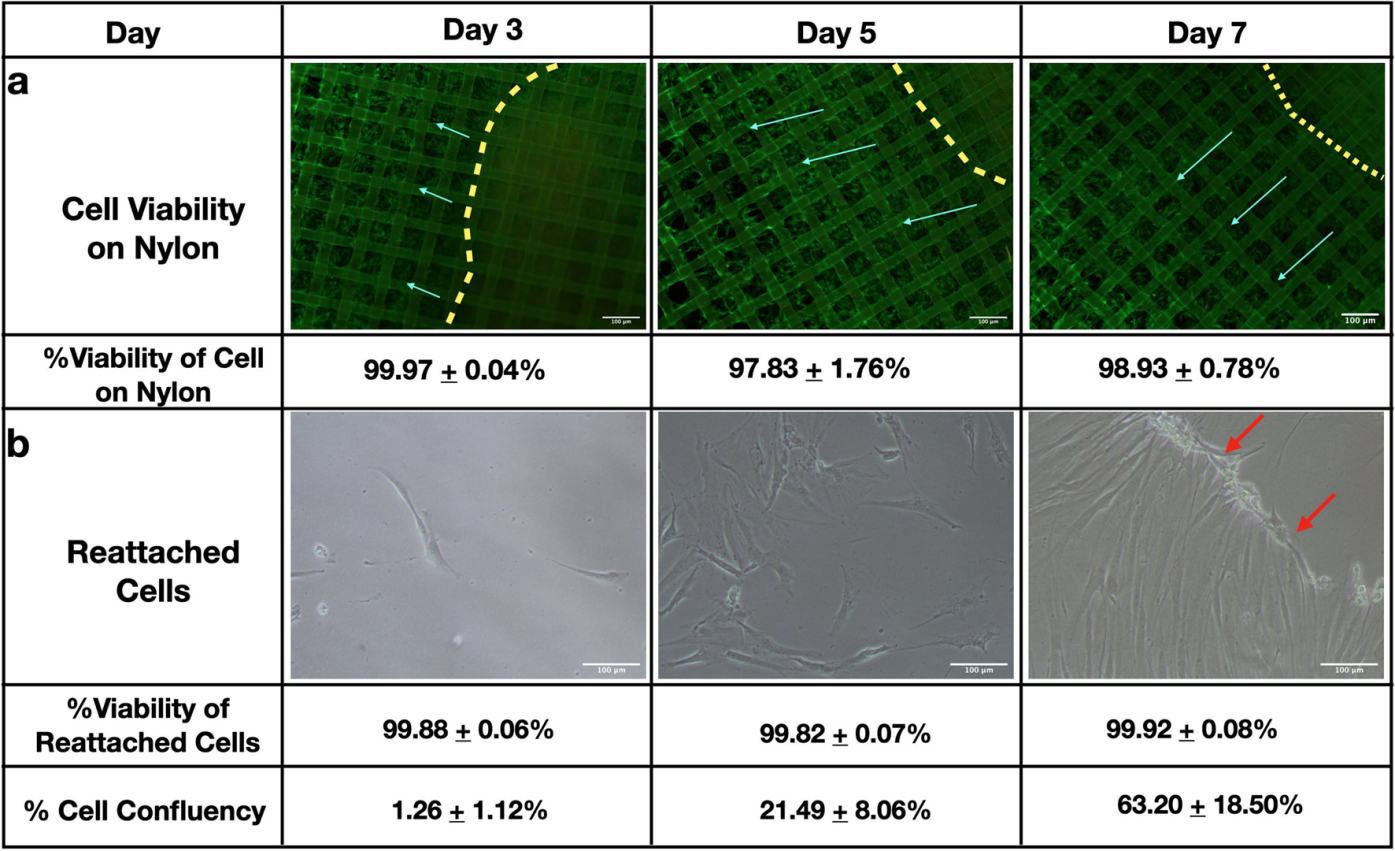
Viability of fibroblast cell sheets on nylon dressing and cell reattachment. (**a**) Fluorescence images of LIVE/DEAD-stained fibroblast cells on nylon dressing at days 3, 5 and 7 and their cell viability percentages are presented. Note that the green fluorescence represents live cells, while red fluorescence indicates dead cells. (**b**) The phase-contrast images show the morphology of the fibroblasts relocated to a new culture dish surface at various time points. The confluency and viability percentages of the reattached fibroblast cells were calculated and are shown in this figure. The yellow dotted line represents the edge of the fibroblast cell sheet, and the light blue arrows indicate the outgrowth cell direction on the nylon dressing. The red arrows show the area where the cell grew out from the edge of the sheet. The results are displayed as the mean ± SD (n = 6). The scale bars represent 100 μm.

Since a fibroblast monolayer sheet is fragile and can be folded easily, the possibility of using nylon dressing as a support to transfer the cell sheet and place it on a wound was explored. Nylon dressing is a good candidate for this application, as it has already been routinely used as a wound dressing material. When dry nylon dressing was placed on top of the detached fibroblast cell sheet, the whole cell sheet immediately attached to the gauze with no further shrinkage (Fig. 1c). The fibroblast cells were allowed to grow on the nylon dressing for 7 days to determine whether the nylon material could support the growth of fibroblasts. The observation was limited to 7 days to avoid possible contact inhibition-induced growth arrest and cell death due to the high density of the fibroblast cells on the nylon dressing. According to Fig. 2, confluent cells with spindle morphology were observed outside the yellow dashed line, which represented the initial edge of the cell sheet, and the arrows indicated the direction of the outgrowth cells. This evidence suggests fibroblast outgrowth from the cell sheet into the adjacent nylon dressing area. In addition, the cell viability of the outgrowth cells remained high, close to 100%, throughout the experiment, indicating that the cells were healthy and active. Moreover, the reattachment ability of the fibroblast cell sheets from the nylon dressing adhering to a tissue culture surface was also analyzed. As shown in Fig. 2, very few fibroblast cells, approximately 1% confluency, were found to migrate from the nylon dressing initially, but after 7 days the number of reattached cells continuously increased to over 60%, having high cell viability and correct morphology. Interestingly, on day 7, most of the reattached cells migrated out of the sheet at the edge, in which the red arrows show the edge of the cell sheet before the dressing was removed. Hence, the nylon dressing could be used as a structural support for cell attachment, growth and migration.

### Cell viability after application of the MEEK method

**Figure 3:**
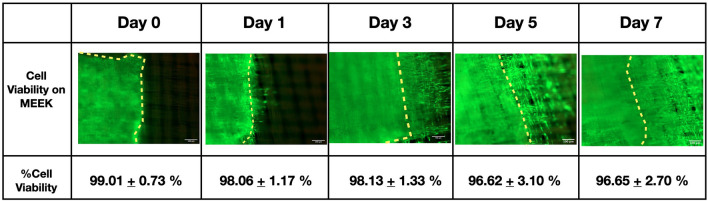
Cell viability of fibroblasts on 1:9 MEEK gauze. The fibroblast cell islands were stained with LIVE/DEAD stain on days 0, 1, 3, 5 and 7, in which green and red fluorescence refer to live and dead cells, respectively. The cell viability was determined from an image analysis (n = 3). The yellow dotted lines indicate the edge of the fibroblast sheet island. The results are displayed as the mean ± SD (n = 10). The scale bars represent 100 μm.

Based on the MEEK technique, the samples must be cut and stretched to create numerous 3 × 3 mm square cell islands. Applying this technique to the cell sheets may cause damage to the cells, affecting cell migration and proliferation. Therefore, the viability of fibroblasts on MEEK gauze after being expanded was examined by using a LIVE/DEAD staining kit, in which green fluorescence indicated live cells, while red fluorescence indicated dead cells, and their intensities were quantified over 7 days. We only evaluated the cell viability on the MEEK gauze at an expansion ratio of 1:9, as a representative for all the expansion ratios. Figure 3 shows the intense green fluorescent cells inside the yellow boundary which indicated the presence of cell sheet islands, while the more dispersed green cells were observed outside the yellow boundary, representing the outgrowth areas. The viability of the fibroblast cells was found to be over 95% at all time points, regardless of the cutting and stretching process. These fibroblast cells on the MEEK gauze migrated and proliferated outside the cell sheet island and occupied the surrounding areas. As a result, MEEK gauze has also been shown to support fibroblast migration and proliferation.

### Cell reattachment of the cell sheet on MEEK at 1:3, 1:6 and 1:9 expansion ratios

**Figure 4:**
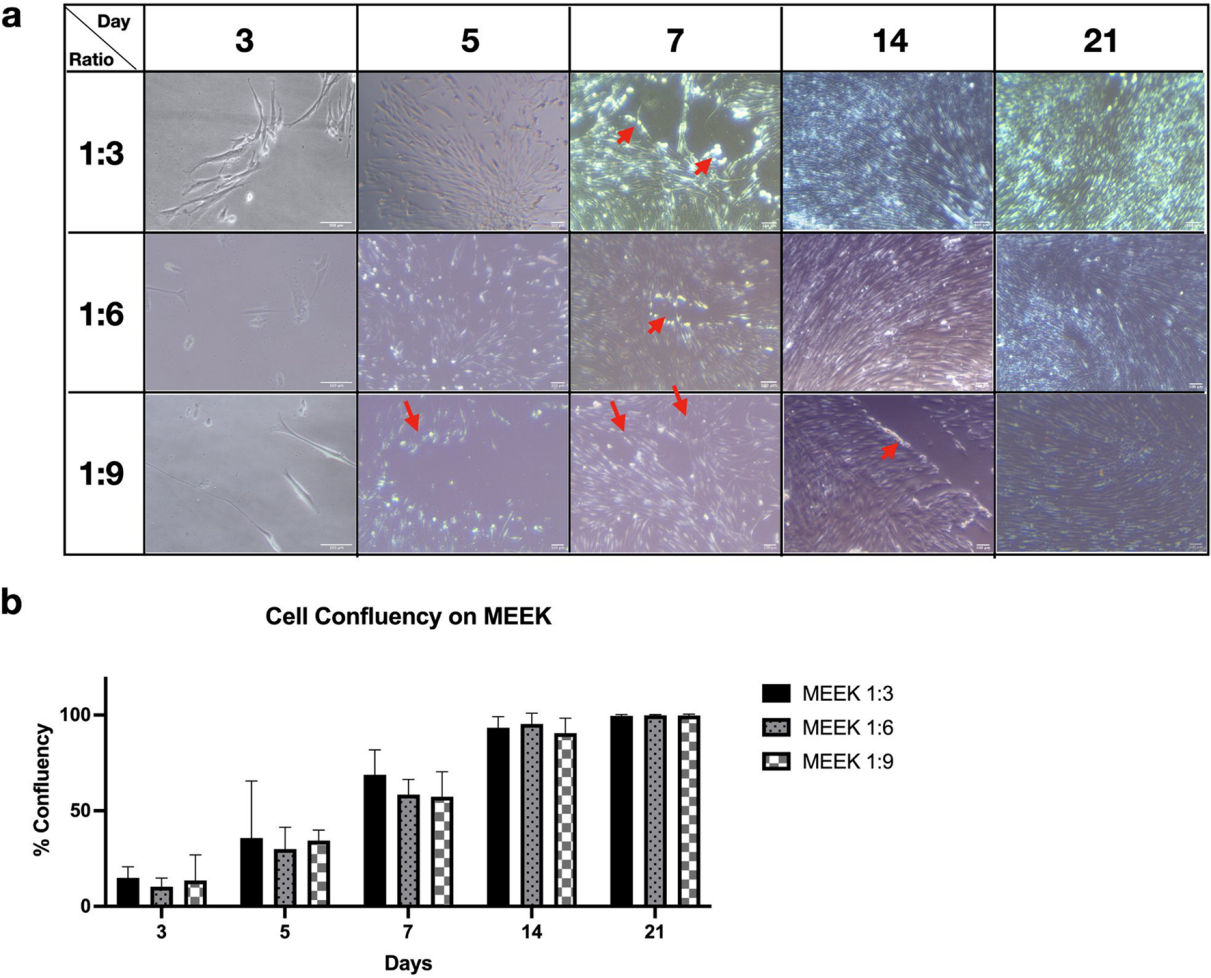
Reattachment of fibroblast cells from MEEK gauze to new culture dishes. (**a**) The presence of fibroblast cells from MEEK gauzes at 1:3, 1:6 and 1:9 expansion ratios was observed at days 3, 5, 7, 14 and 21 under a phase contrast microscope. The red arrows indicate the edges of the cell sheet islands. (**b**) The confluency of fibroblasts on new surfaces at various time points was determined. The results are displayed as the mean ± SD (n = 6). Statistically significant differences were defined using one-way ANOVA with Tukey’s multiple comparison test. The scale bars represent 100 μm.

In the typical MEEK micrografting technique, autologous skin islands on MEEK gauze are normally transferred and grafted to the wound to facilitate re-epithelialization. To prove that this concept was also true for the cell sheets on MEEK gauze, we investigated the ability of the fibroblast cells on MEEK gauze to translocate and reattach to a new culture dish surface. Within 3 days, the cells migrated from the cell sheet islands and reattached onto the new culture surface, and those cells continuously proliferated. As shown in Fig. 4a, on day 7, many more cells were found on the surface and had spread to most areas. Clearly, the cells migrated from the edge of the previously occupied cell sheet islands, indicated by the red arrows. On day 14, the reattached fibroblast cells became more confluent and denser, occupying the empty spaces. On day 21, the cells grew and migrated through the inside sheet island area, providing the whole culture surface coverage. Cell confluency was determined and is shown in Fig. 4b. The average cell confluency was initially lower than 20% but continuously increased to 100% by day 21. However, the differences in confluence between the different expansion ratios were not statistically significant. This result shows that MEEK gauze could allow cell reattachment and enable prolonged cell cultivation, providing high confluency within 21 days.

### Cell migration of fibroblast sheets on the nylon dressing and MEEK gauze at 1:3, 1:6 and 1:9 expansion ratios

**Figure 5:**
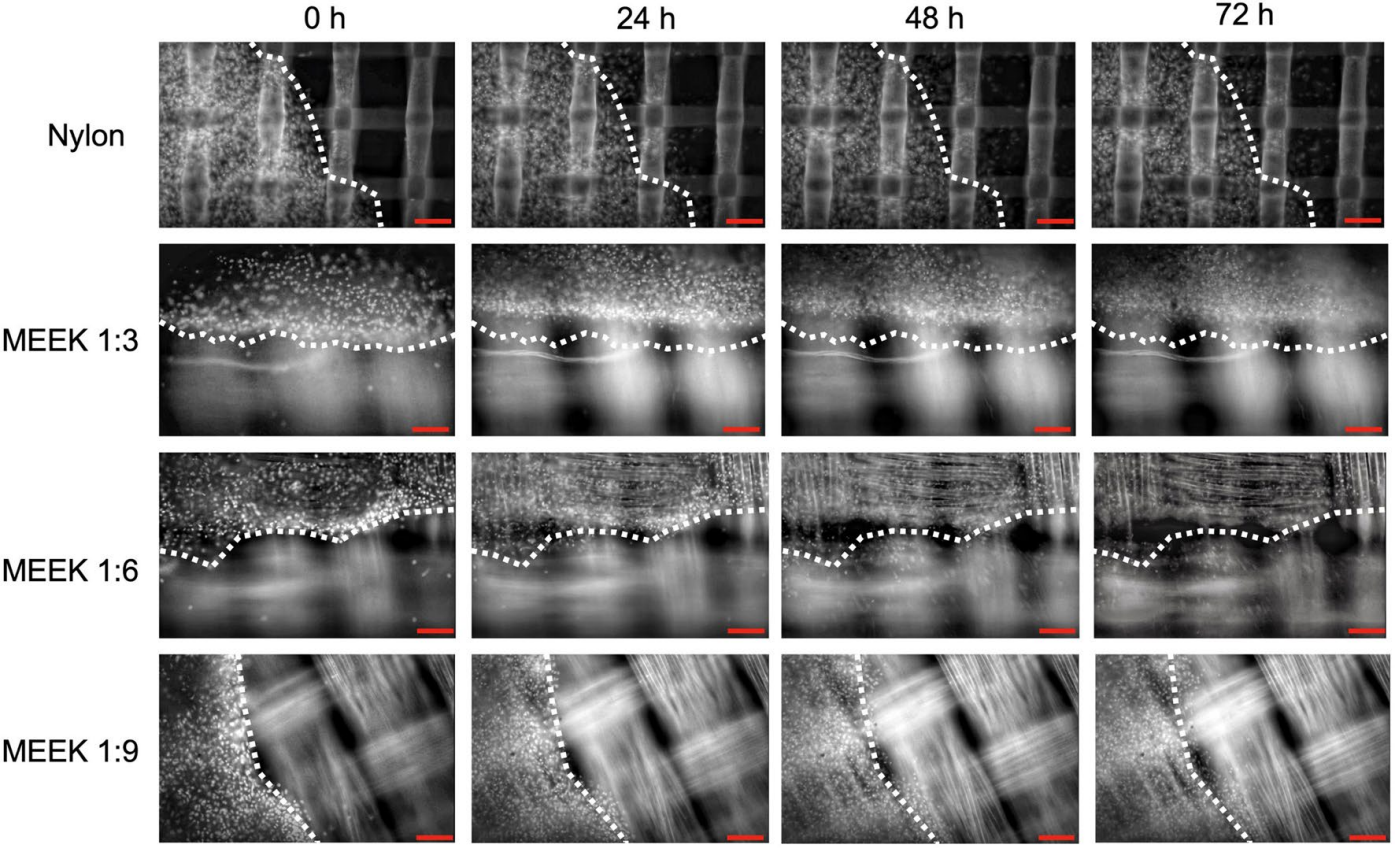
Fibroblast migration on both nylon dressing and MEEK gauzes. Fibroblast movement was shown at 0, 24, 48 and 72 h on nylon dressing and MEEK gauzes at 1:3, 1:6 and 1:9 expansion ratios. The dotted lines indicate the initial edge of the cell sheet or cell sheet island. The scale bars represent 100 μm.

Cell migration on nylon dressing and MEEK gauze was investigated, as it is the fundamental process of tissue homeostasis and wound healing. The fibroblast migration patterns on the nylon dressing and MEEK gauzes at expansion ratios of 1:3, 1:6 and 1:9 are shown in Fig. 5, where the dotted lines indicate the initial edge of the cell sheets on the nylon dressing or the cell sheet islands on MEEK gauzes. Since the cell sheets on the MEEK gauzes were cut into smaller islands, clear straight-line edges with dense cells were observed. Unlike the MEEK technique, the cell sheet was directly placed onto the nylon dressing, showing a gradient of cell density around the periphery. Consequently, the cells on the nylon dressing could immediately migrate along the fiber toward less crowded areas with elongated morphology, as shown in Fig. 5 and Supplementary S1. On the MEEK gauze, the fibroblasts mostly remained stationary during the first 24 h but moved forward from the periphery of the cell sheet islands along the fiber of the MEEK polyamide gauze, with the cells on the 1:6 MEEK gauze having the furthest migratory distance (Fig. 5 and Supplementary S2).

### Comparison of the average velocities and trajectories of the cells on nylon dressing and MEEK gauzes

**Figure 6:**
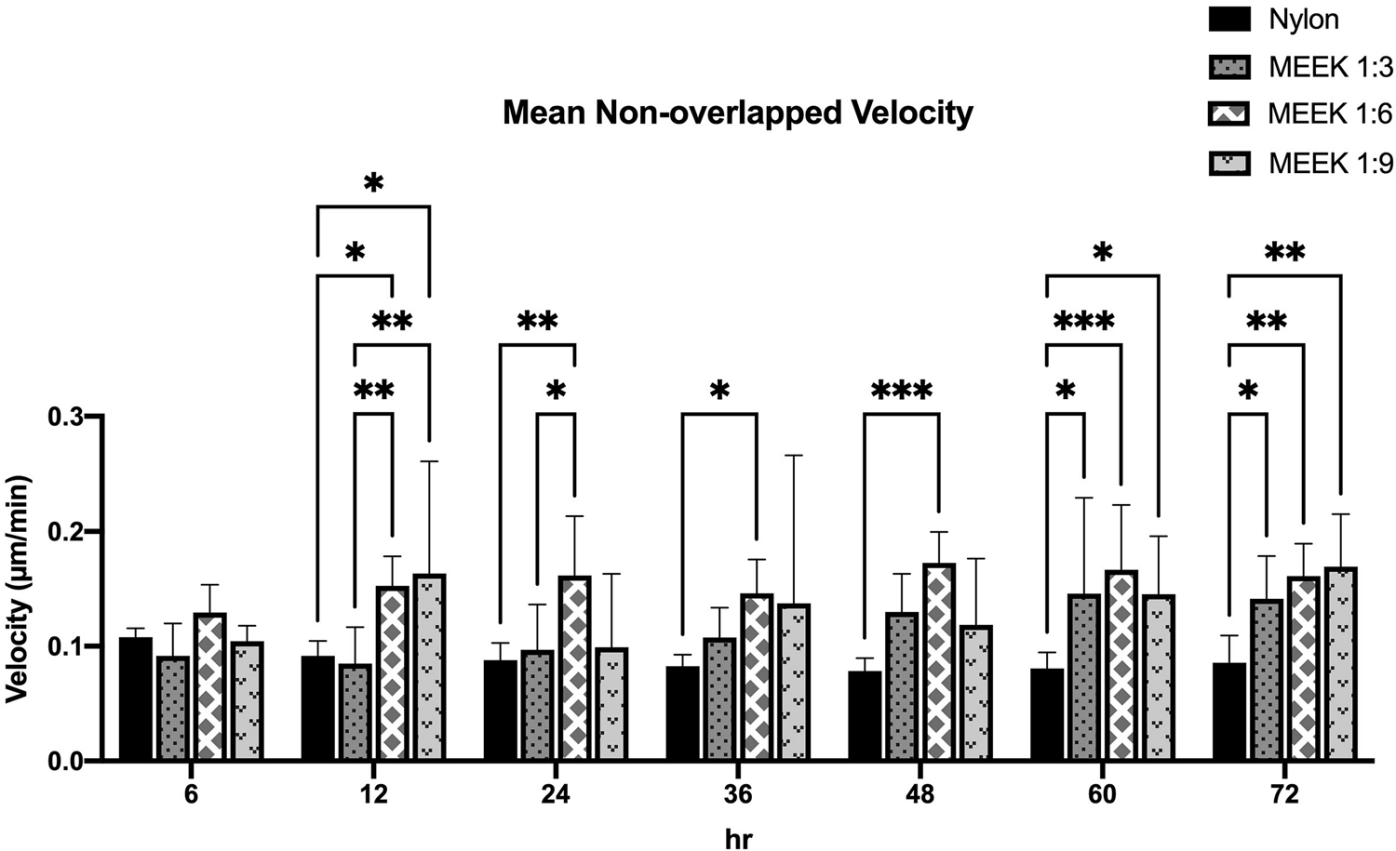
Mean nonoverlapping velocity of fibroblast cells on nylon dressing and MEEK gauzes. The migration velocities of fibroblasts on nylon dressing and MEEK gauzes at 1:3, 1:6 and 1:9 expansion ratios were determined at 6, 12, 24, 36, 48, 60 and 72 h. The results are shown as the mean ± SD (n = 5). Statistically significant differences were determined using two-way ANOVA with Tukey's multiple comparisons test, in which *p < 0.05; **p < 0.01, and ***p < 0.001.

**Figure 7:**
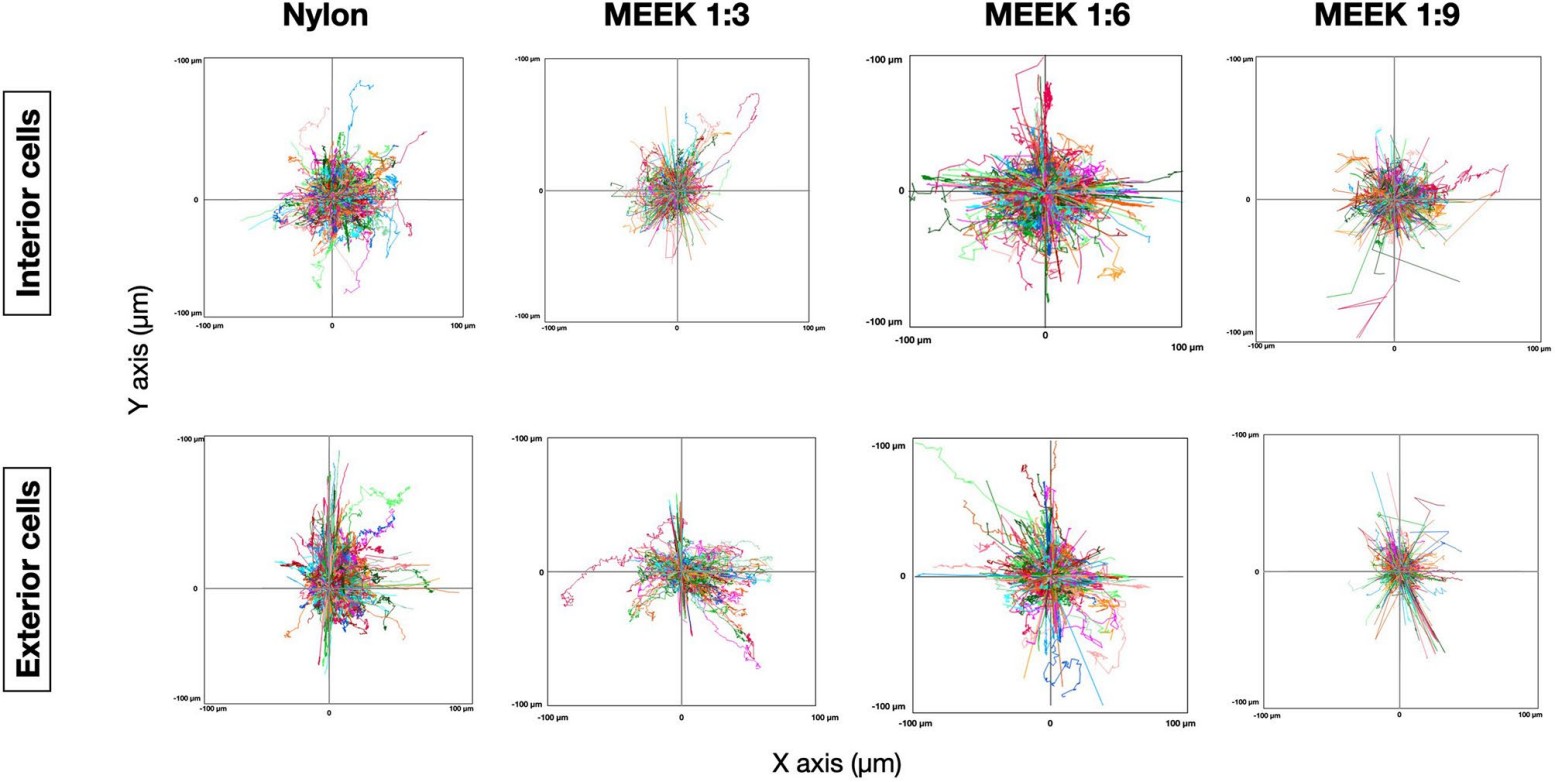
Wind-Rose plots depicting the migratory behaviors of fibroblast cells on various materials. The fibroblast cells on nylon dressing and MEEK gauzes at 1:3, 1:6 and 1:9 expansion ratios were stained to track their movement, and the cell trajectory was analyzed in each condition. The trajectory plot represents the cell migration direction and distance at the interior and exterior of both nylon dressing and MEEK gauzes during 0–72 h. Each line indicates an individual path of each cell.

The average velocities of the cells on nylon dressing and MEEK gauzes at expansion ratios of 1:3, 1:6, and 1:9 at various time points are shown in Fig. 6. The mean nonoverlapping velocities of the cells at 6 h were low and similar in all groups. From 12 to 48 h, the average velocities of the cells on the MEEK gauze at an expansion ratio of 1:6 were the highest. From 60 to 72 h, the average velocities of the cells on the nylon dressing remained constant, while those of the cells in other conditions noticeably increased. The migratory paths of the fibroblasts on all gauzes were tracked and are shown as wind-rose plots in Fig. 7. Each color line represents the total path of a single fibroblast over 72 h. As shown in Fig. 7, the larger diameters of the wind rose plots were observed from the fibroblast cells at the interior and exterior on the MEEK gauze at a 1:6 ratio, indicating the enhanced motility of these cells at this expansion ratio. The fibroblast cells on other surfaces had noticeably shorter migratory paths.

## Discussion

The major obstacle in using skin cell sheets in clinical practice is their size limit^5^. In real applications, skin cell sheets need to be at least ten times larger than their current size for full wound area coverage to promote effective treatment^6,7^. To overcome this issue, we first considered constructing a larger cell sheet by using a larger surface area because the area of the temperature-responsive culture surface directly affects the size of the harvested cell sheets. However, with an increased temperature-responsive culture surface size, a significantly large number of cells were required to obtain the same seeding density, which was very labor intensive and prone to technical errors. We, therefore, modified our protocol by reducing the cell density and allowing the cells to proliferate for a few days until confluence. Unfortunately, by increasing the cell culture time on the temperature-responsive polymer, the cells attached too strongly to the surface, making cell detachment as an intact sheet challenging. During the cell sheet harvesting process, the cell sheet was broken into smaller pieces (Supplementary S3). Thus, we proposed the use of nylon dressing or MEEK gauzes as cell sheet supporting material during cell sheet harvesting and for cell sheet expansion. Nylon is an inexpensive material and is currently used as a wound dressing, while MEEK prefolded gauze is extensively used in severe burn patients, giving a high re-epithelialization rate^12,17,19^. To verify their potential application with cell sheet engineering, we investigated the qualities and behavior of fibroblast cell sheets on both fabrics.

In this work, human primary dermal fibroblasts were used to closely mimic the in vivo state and physiology. Unfortunately, the use of primary cells in biomedical research has several drawbacks. One main challenge of primary cells is that cells taken from different donors might behave differently. To overcome this problem, primary fibroblasts used in all experiments were isolated from the same donor to ensure consistency and reliability of the cell behaviors. As only a limited number of cells could be isolated from skin samples of one donor and a large number of cells were required to maintain tight cell–cell adhesion during the cell sheet detachment process, a higher number of cell passages (up to passage 9, but mostly passages 6–7) were used in our experiments. Generally, for primary culture, passages 2–5 are recommended to maintain the cells’ genetic and phenotypic properties without becoming senescent. However, according to our previous work, primary fibroblasts at passage 10 still possessed high cell viability and preserved important cellular functions such as proliferation, migration and cytokine secretion^5^. Some other studies also reported the use of primary human dermal fibroblasts at passage 10 in their investigation^20,21^.

Our results showed that the cell sheets could firmly attach to the nylon dressing and MEEK gauze without the use of adhesives (Figs. 3,4). More importantly, the fibroblast cell sheets on the MEEK gauze did not detach from the gauze, even after being cut and stretched. The strong attachment of the cell sheets to both materials was possibly due to the adhesive protein that was harvested together with the cell sheets^22^. Normally, in the MEEK technique for skin grafts, the proprietary glue Humeca® glue is required to keep the donor skin sample in place during the cutting process and is critical to the outcome of skin graft transfer^23^. Fortunately, with the adhesive protein harvested with the cell sheet, Humeca® glue was unnecessary. This would be particularly beneficial in reducing the preparation steps in the protocol and there would be no concern over the effect of the synthetic adhesive glue on the cell viability and migration.

In addition to cell attachment, both the nylon and MEEK fabrics were biocompatible with the cells and could clearly support cell growth. Hence, we can assume that even though cutting the cell sheets might have caused cell damage around the edges, it did not have any effects on cell proliferation and migration. According to our previous study, fibroblast cell sheets could help accelerate the wound healing process by releasing essential cytokines and growth factors that regulate wound repair^5^. A higher cell number would lead to more cytokine and growth factor secretion, possibly leading to faster wound healing. According to Fig. 4b, even though fibroblasts could be cultured for up to 21 days, it would be more practical to limit the use of this cell-based wound dressing to 14 days, as the cell confluency was close to 100%. After 14 days, the overconfluent cells would undergo contact inhibition, causing growth arrest and possibly diminishing the therapeutic effects of the dressing.

The migration patterns between the fibroblasts on the nylon dressing and MEEK gauze were quite similar, as the cells migrated from the periphery of the cell sheets and preferentially moved along the matrix fibers (Fig. 5). The aligned fibers could induce cellular elongation and the alignment of collagen secretion by fibroblasts, guiding the migration of the cells to the defect area^24,25^. According to the migration movie clips (Supplementary S2), individual fibroblasts initially detached themselves from the monolayer and moved away in a noncoherent pattern but were directed toward free space. The migration of fibroblasts occurring earlier on nylon dressing, compared to MEEK gauze, was possibly the result of the lower cell density at the periphery of the cell sheets. At a lower cell density, stronger cell–substrate interactions overcome cell–cell interactions, allowing the cells to escape the monolayer^26^. On the other hand, in all MEEK conditions, single cells took longer to detach from the dense cells at the edge of the monolayer due to strong cell–cell interactions, leading to no immediate net movement at earlier time points. After 24 h, more cells were observed outside the edge of the cell sheets, possibly due to the combination of cell migration and proliferation.

Our results showed that the migration rates of fibroblasts on the MEEK gauze were predominantly higher than those on the nylon dressing. Even though both fabrics were made of the same polymer, which is polyamide, the topography of the substrates and densities of the fibers were clearly different. These factors have been previously shown to affect cell-substrate adhesion properties^27^, which directly influences cell motility^28^. In addition, cutting the cell sheets into small cell sheet islands in the modified MEEK technique resembled wounding the cell monolayer in a scratch assay, which was reported to affect cell migration by inducing changes in gene expression and signaling^29^. There is evidence that cells produce chemicals or signals, such as ATP or Ca^2+^ after injury^30,31^. Increases in the levels of ATP and Ca^2+^ have been shown to enhance and stimulate fibroblast proliferation, migration, and ECM production which are involved in wound healing mechanisms^30^. Another possible explanation for the higher migration rate of fibroblast cells on the MEEK gauze is mechanical stimulation due to the stretching of the MEEK gauze for cell sheet expansion. Stretched fibroblasts have been reported to migrate faster and move a farther distance than their nonstretched counterparts^32^. Stretching caused the upregulation of matrix metalloproteinases (MMPs), which are responsible for collagen degradation, leading to lower cell–substrate interactions and resulting in increased migration^32^. Our result is also consistent with a previous report that autologous micrografts could enhance the cell migratory capacity, resulting in accelerated wound closure in vitro and in vivo^33^.

The main difference between MEEK gauzes at different expansion ratios is the spacing between the cell islands, in which the expansion ratios of 1:3, 1:6 and 1:9 yielded 2.2 mm, 4.5 mm, and 6 mm between cell islands, respectively^34^. The migration rate of fibroblasts on the MEEK gauze at a 1:6 expansion ratio was found to be the highest, indicating that the distance between cell islands had an effect on the movement kinematics of the cells. A previous study by Toume et al. reported a linear relationship between the initial gap area and the fibroblast migration rate, in which the larger the initial gap size was, the higher the migration rate^35^. Thus, the lowest migration rate observed from the cells on 1:3 expanded MEEK gauze was probably the result of the shortest distance between each cell island at this particular expansion ratio. In addition, using an expansion ratio of 1:3 may not be significantly beneficial, as it can only cover a small wound area, which commonly heals quite quickly^17^. Interestingly, the migration rate of fibroblasts on the MEEK gauze at a 1:9 expansion ratio was lower than that of the cells on the MEEK gauze at a 1:6 expansion ratio. It was possible that the gap distance between each cell island on 1:9 expanded MEEK gauze might have been too far for cell–cell communication and signaling. Generally, cells communicate with each other through the release of soluble cytokines and chemokines. These signaling molecules diffuse through the medium, bind to the cell’s receptors and activate many crucial biological pathways responsible for proliferation, migration, etc^36^. The longer distance between each cell island on the MEEK gauze at a 1:9 expansion ratio would have led to a larger diffusion length and possibly lower cytokine concentrations to induce cell migration. Note that the interpretation of our findings was mainly based on the previous studies. To validate the above explanation, a thorough investigation is needed. Nonetheless, the expansion ratio of 1:6 was shown to be optimum, resulting in a faster cell migration, which could potentially lead to accelerated wound healing in large chronic and burn wounds.

In conclusion, we have shown that the combination of the fibroblast cell sheet with nylon dressing or MEEK gauzes at various expansion ratios may overcome the limitation of the cell sheet applications in clinical settings, including limited treatment area and cell sheet handling due to its fragility. Fibroblast cells on nylon dressings and the expanded fibroblast sheets on MEEK gauzes have been shown to possess high capacities of cell proliferation and migration which could be beneficial in wound care applications, particularly for nonhealing chronic wounds or injuries from significant skin loss. This combination technique can be applied to different cell types, such as keratinocytes and endothelial cells, to construct large cell sheets and apply them to wounds to speed up the re-epithelialization^37^ and vascularization processes^38^. The enlargement of multilayered co-culture cell sheets, including keratinocyte-fibroblast or fibroblast-endothelial cells, using MEEK micrografting would also be worth investigating, as the wound healing potential of the co-culture system has been shown to be superior to that of a single cell type tissue^39^. Note that internal separation or delamination in the multilayered cell sheets was previously reported^40^. It is possible that the cell sheet layers could be separated upon stretching to the desired expansion ratio. Therefore, further study is needed to identify the limit of the cell layers and the combination of cell types that can be used with the MEEK micrografting technique.

## Methods

### Dermal human fibroblast cell isolation

The collection of discarded split thickness skin samples and all experimental protocols were approved by the Human Research Protection Unit, Faculty of Medicine, Siriraj Hospital, Mahidol University (Si 587/2017). All methods were carried out in accordance with relevant guidelines and regulations. Informed consent was obtained from all subjects and/or their legal guardians. The discarded skin samples were obtained from one patient aged 26 years old with a total burn surface area (TBSA) of less than 30%. Patients diagnosed with diabetes mellitus, skin diseases and bloodborne infectious diseases including hepatitis B, hepatitis C, and human immunodeficiency virus (HIV) were excluded from this study. Fibroblast cells were isolated as previously described^7^. Briefly, a split-thickness skin sample was cut into 1 cm wide strips, and the skin was immersed in 2.4 U/mL Dispase II (Invitrogen, Carlsbad, CA, USA) overnight at 4 °C to disassociate the connective tissue. The epidermis or darker layer was peeled from the skin, leaving only the dermis layer, which was finely chopped and kept in Trypsin/EDTA 0.25% solution (Invitrogen) for 30 min at 37 °C. Then, the fibroblast cells were collected by centrifugation at 1500 RPM for 5 min. The cell pellet was resuspended in its culture medium, composed of Dulbecco’s Modified Eagle Medium and Ham’s F’12 (Invitrogen), at a 3:1 ratio supplemented with 10% fetal bovine serum (FBS) and 1% antibiotic–antimycotic. The cells were kept in a 5% CO_2_ humidified environment at 37 °C.

### Fabrication of PNIAM-co-AM grafted plates

The fabrication of PNIAM-*co*-AM grafted surfaces followed the method developed by Sakulae et al.^4^. In short, 35 mm polystyrene culture dishes (Falcon 3001, BD Bioscience, Billerica, MA, USA) were exposed to UV light (UVGL-58, 6 W) at 254 nm for 30 min to activate the culture dish surface. All the chemicals used in this procedure were purchased from Sigma–Aldrich (St. Louis, MO, USA), except for acrylamide (Merck, Kenilworth, NJ, USA). A mixture of *N*-isopropylacrylamide (NIAM) and acrylamide (AM) at a 1:1 molar ratio, N,*N'*-methylenebisacrylamide (MBAM), a cross-linker, and potassium periodate (KIO_4_), a photoinitiator, was added and spin-coated onto the UV-activated culture dish at 1500 RPM for 5 min. Then, the surface was immediately exposed to 254 nm UV for 2 h. Afterwards, the polymerized surface was rinsed with 70% ethanol to remove unreacted monomers and dried in a vacuum oven at 30 °C for 24 h^3,4^. To sterilize the PNIAM-*co*-AM grafted dishes for cell culture use, these dishes were washed with 70% ethanol, followed by rinsing with phosphate buffered saline (PBS, Invitrogen) 3 times.

### Cell sheet construction using the PNIAM-co-AM grafted plates

The construction of a monolayer cell sheet has already been described elsewhere^7^. Human fibroblast cells at passages 6–9 were seeded onto 35 mm PNIAM-*co*-AM grafted dishes at a density of 3.5 × 10^5^ cells/cm^2^ and incubated at 37 °C for 24 h to allow the cells to firmly attach to the surface. After that, the incubation temperature was reduced to 10 °C for 30 min and later increased to 20 °C for 60 min to allow the cells to detach from the surface as an intact cell sheet. The cell sheet was transferred to a new culture dish by pipetting to unfold and straighten out the cell sheet before placing it onto gauze.

### Transferring of the cell sheet onto nylon dressing

Nylon dressing (3 M™ Tegaderm™ Contact Layer, St. Paul, MN, USA) was cut into circles with the same diameter as a 35 mm culture dish and sterilized by using an autoclave. After the fibroblast cell sheet was harvested and transferred to a new culture dish, dry circular nylon dressing was overlaid on top of the cell sheet and immediately flipped using forceps to allow the cell sheet to face upward (Fig. 1a,c). Five hundred microliters of the fibroblast medium was gently added to the side of the nylon dressing to prevent the cells from drying before the sample was incubated overnight at 37 °C. The attachment of the fibroblast cell sheet to the nylon dressing was confirmed using a phase contrast microscope.

### Enlargement of cell sheets using the modified MEEK technique

The fibroblast cell sheets were enlarged using a technique modified from MEEK micrografting^8,16^. In this study, only polyamide pleated sheets with aluminum backing or MEEK gauze (Humeca BV, Enschede, The Netherlands) at expansion ratios of 1:3, 1:6 and 1:9 were used. Two fibroblast cell sheets were transferred onto MEEK gauzes using the same procedure as that of the nylon dressing (Supplementary S4a). After the cell sheets were well positioned on MEEK gauzes, they were cut vertically and horizontally by hand using a surgical blade, following the previously marked pleats on the gauze. To separate each cell sheet square island, the MEEK gauze was stretched on all four sides until the gauze completely unfolded and the aluminum backing was removed (Fig. 1b,d and Supplementary S4b,c). To examine cell reattachment and migration, the expanded MEEK gauzes with cell sheet islands were cut into rectangles to fit with 35-mm culture dishes (Supplementary S4d). Next, the cell sheet islands on the MEEK gauze were maintained for 24 h in a small volume of fibroblast culture medium to prevent the cell sheets from floating upward.

### Cell viability after transfer to nylon dressing and MEEK gauze

The viability of fibroblast cells on the nylon dressing (n = 6) and on the expanded MEEK gauze (n = 10) was analyzed using a LIVE/DEAD staining kit (Invitrogen, Carlsbad, CA, USA) over 7 days. At predetermined time points, the cells were stained with a LIVE/DEAD solution comprising Calcein-AM and ethidium homodimer-1 (EthD-1), according to the manufacturer’s instructions. Afterwards, the fluorescence of the stained cells was observed using Cytell™ Cell Imaging System (GE Healthcare, Arlington Heights, IL, USA) at excitation/emission wavelengths of 485/530 nm and 530/645 nm for Calcein-AM and EthD-1, respectively. Its fluorescent intensities were quantified by ImageJ software (NIH, Bethesda, MD, USA).

### Transfer of fibroblast cell sheets from nylon dressing and MEEK gauze to new surfaces

The cell sheets on the nylon dressing and MEEK gauze were turned upside down and placed onto new 35 mm tissue culture dishes to allow the cells to reattach (Supplementary S4d). Two milliliters of the culture medium was added to these dishes to maintain cell growth. The medium was changed every 2–3 days. At predetermined time points, the gauze was removed from the culture dish before observation using a phase-contrast microscope. In the nylon sample, the translocated cells were investigated on days 3, 5 and 7, while the observation of the cells from MEEK gauze was prolonged to 14 and 21 days. The confluency of the reattached cells (n = 6) was analyzed by ImageJ software in the area where the cells grew out and outside the gauze coverage.

### Determination of the cell migration, velocity, and trajectory on nylon dressing and MEEK gauze

To track cell movement, the fibroblast cells on the gauzes were initially stained with NucBlue™ Live ReadyProbes™ Reagent (Molecular Probes, Waltham, MA, USA) at 20 °C for 20 min. Then, the stain solution was removed and 2 µl of fresh medium was added to maintain the cell culture during evaluation. The cell sheet samples on the gauze were placed on tissue culture dishes with the cell sheets facing down. Afterwards, time-lapse images of the cell migration on the gauze were acquired on Biostation IM-Q microscope (Nikon Inc, Melville, NY) with a 10X magnification (numeric aperture 0.5) and taken every 10 min at various spots for 72 h at 37 °C in a 5% CO_2_ humidified atmosphere. The determination of the mean nonoverlapped velocity (n = 5) and trajectory of the cells were analyzed using CL-Quant software version 3.30 (Nikon Corporation, Minato-ku, Tokyo, Japan).

### Statistical analysis

All the results are presented as the mean ± SD. Two-way ANOVA followed by Tukey’s multiple-comparison tests (GraphPad Software, La Jolla, CA, USA) was applied to analyze the difference between groups of data in the cell migration study, while one-way ANOVA was used to analyze the data from the other experiments. A statistically significant difference was considered when the P value was less than 0.05.

## Supplementary Information

Supplementary Video 1.

Supplementary Video 2.

Supplementary Video 3.

Supplementary Video 4.

Supplementary Information 1.

## Data Availability

All data generated or analyzed during this study are included in this published article and its supplementary files.
