# Supplementary material for: Expansion of fibroblast cell sheets using a modified MEEK micrografting technique for wound healing applications
Source: Sci Rep. 2022 Nov 3;12:18541. doi: 10.1038/s41598-022-21913-x (PMC9633782; doi:10.1038/s41598-022-21913-x)
Supplement: Supplementary file 5 — Supplementary Information 1. [file 41598_2022_21913_MOESM5_ESM.docx]

**Supplementary Information**


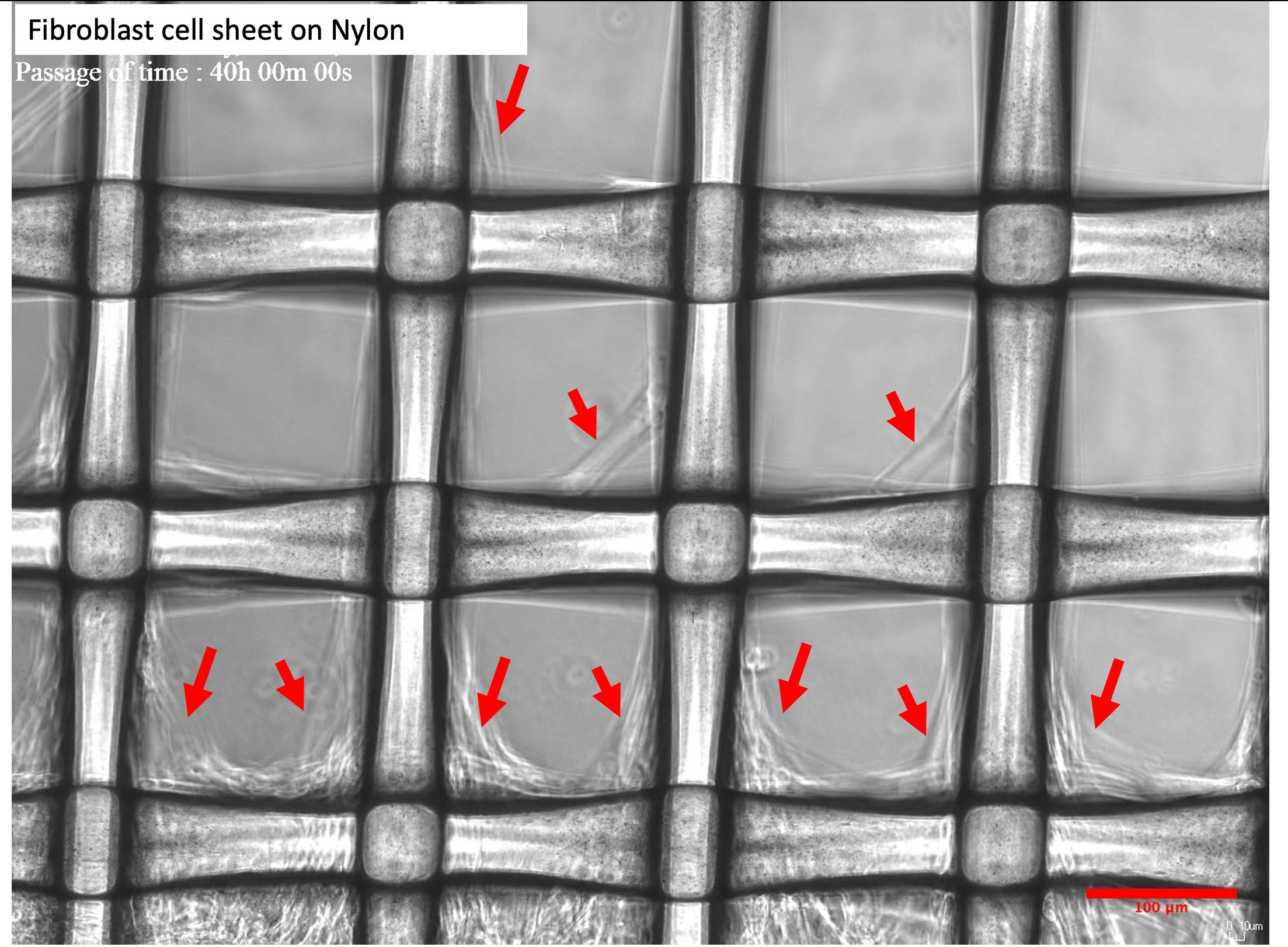


**Figure. S1 Fibroblast migratory behavior on nylon dressing.** Fibroblasts moved along the nylon fiber, as indicated by the red arrows. The scale bar shows 100 μm.

**VDO files**

**S2 Time-lapse video clips of fibroblast migration on nylon dressing and MEEK gauzes at 1:3, 1:6 and 1:9 expansion ratios.** Before observation, the cells were stained with NucBlue™ Live ReadyProbes™ Reagent to track the cell location. All the video clips were recorded from 0 – 72 h. The scale bars show 10 μm.


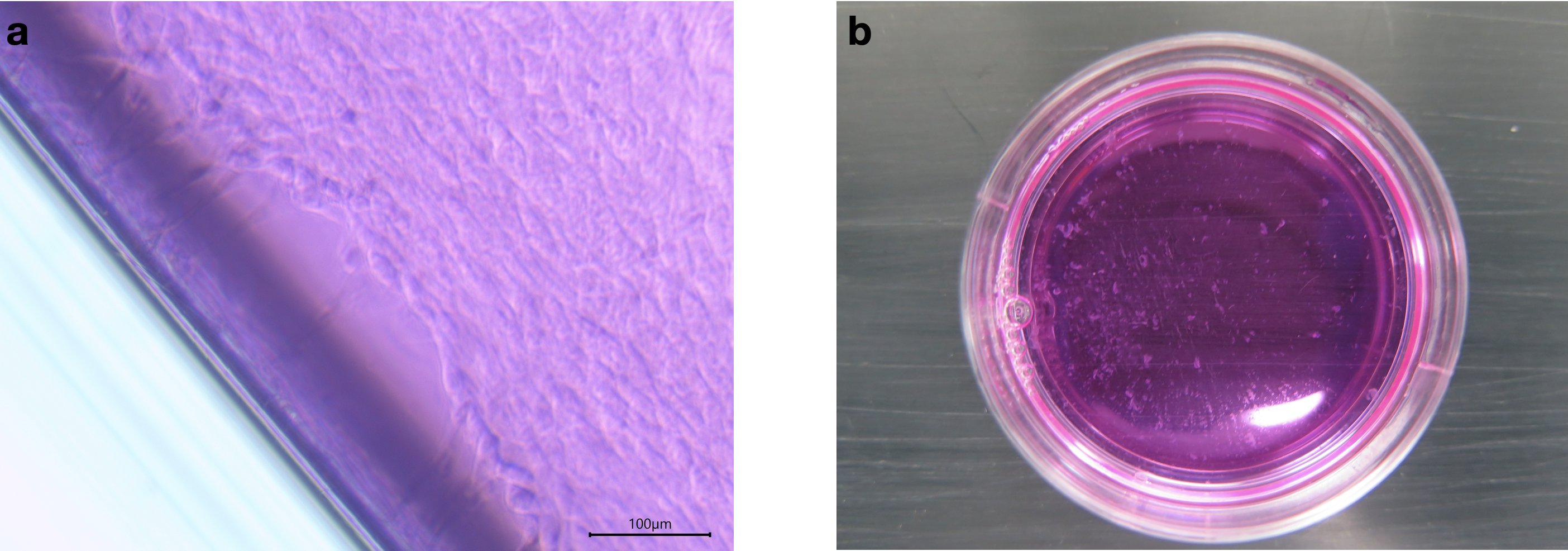


**Figure. S3 Preliminary experiment on cell sheet expansion using a larger temperature-responsive tissue culture surface.** Fibroblast cells were cultured on a temperature-responsive PNIAM-co-AM grafted culture dish with 35 mm diameter. After temperature reduction, the cell sheet broke into small pieces. The scale bar is 100 μm.


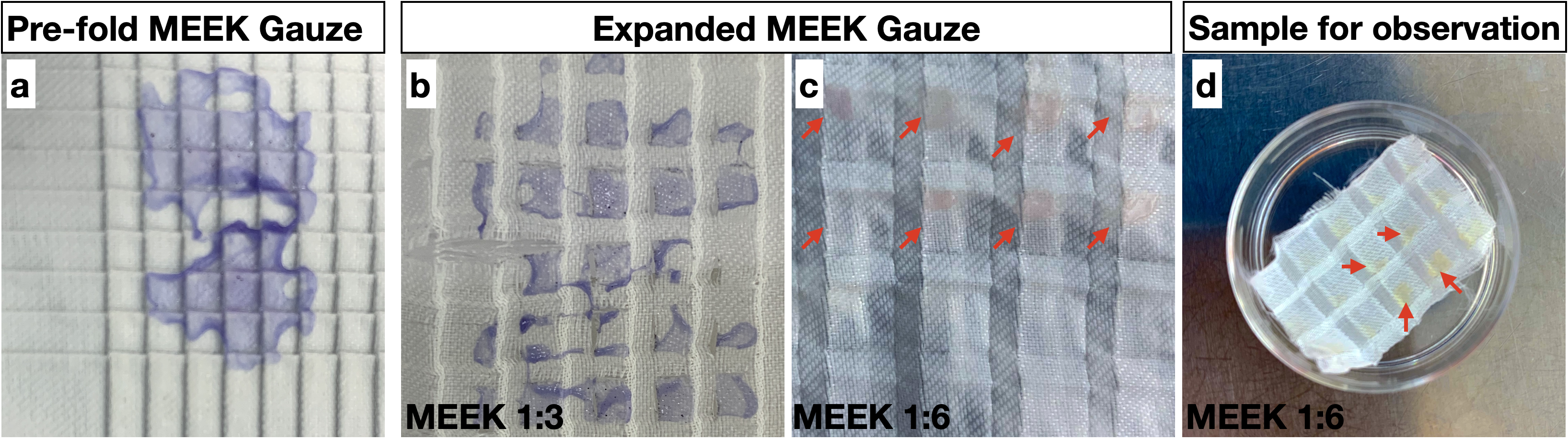


**Figure S4:** **The process of expanding cell sheets in the MEEK groups.** (a) The fibroblast cell sheets harvested from the temperature-responsive culture surface, were overlaid on the pre-fold MEEK gauze. (b) After cutting the cell sheets along the pleats using a surgical blade, the gauze was stretched in all directions to create numerous square islands of cell sheets. The MEEK gauze at different expansion ratios resulted in different spacings between each cell island. (c) The MEEK gauze with the cell sheets was cut to 32.5 mm × 17 mm rectangles to fit with a 35-mm culture dish for observation. The red arrows indicate the cell sheet islands.
